# Chemical Composition of Essential Oils from Natural Populations of *Artemisia scoparia* Collected at Different Altitudes: Antibacterial, Mosquito Repellent, and Larvicidal Effects

**DOI:** 10.3390/molecules29061359

**Published:** 2024-03-19

**Authors:** Amna Parveen, Muhammad Ghazanfar Abbas, Ken Keefover-Ring, Muhammad Binyameen, Raimondas Mozūraitis, Muhammad Azeem

**Affiliations:** 1 Department of Chemistry, COMSATS University Islamabad, Abbottabad Campus, Abbottabad 22060, Pakistan; amna.akbar110@gmail.com; 2 Laboratory of Insect Chemical Ecology, Department of Entomology, Faculty of Agricultural Sciences & Technology, Bahauddin Zakariya University, Multan 60800, Pakistan; ghazanfarentomologist@gmail.com (M.G.A.); mbinyameen@bzu.edu.pk (M.B.); 3 Department of Botany, University of Wisconsin-Madison, Madison, WI 53706, USA; ken.keefoverring@wisc.edu; 4 Department of Geography, University of Wisconsin-Madison, Madison, WI 53706, USA; 5 Laboratory of Chemical and Behavioural Ecology, Institute of Ecology, Nature Research Centre, 08412 Vilnius, Lithuania; 6 Department of Zoology, Stockholm University, Svente Arrhenius vag 18B, 10691 Stockholm, Sweden

**Keywords:** aromatic plants, antibacterial, bioactive compounds, *Aedes aegypti*, pest control, natural remedies, chemotypes, *Artemisia scoparia*

## Abstract

The current study aimed to evaluate the presence of chemical variations in essential oils (EOs) extracted from *Artemisia scoparia* growing at different altitudes and to reveal their antibacterial, mosquito larvicidal, and repellent activity. The gas chromatographic–mass spectrometric analysis of *A. scoparia* EOs revealed that the major compounds were capillene (9.6–31.8%), methyleugenol (0.2–26.6%), β-myrcene (1.9–21.4%), γ-terpinene (1.5–19.4%), *trans*-β-caryophyllene (0.8–12.4%), and eugenol (0.1–9.1%). The EO of *A. scoparia* collected from the city of Attock at low elevation was the most active against *Staphylococcus aureus*, *Bacillus subtilis*, *Escherichia coli*, and *Pseudomonas aeruginosa* bacteria (minimum inhibitory concentration of 156–1250 µg/mL) and showed the best mosquito larvicidal activity (LC_50_, 55.3 mg/L). The EOs of *A. scoparia* collected from the high-altitude areas of Abbottabad and Swat were the most repellent for females of *Ae. aegypti* and exhibited repellency for 120 min and 165 min, respectively. The results of the study reveal that different climatic conditions and altitudes have significant effects on the chemical compositions and the biological activity of essential oils extracted from the same species.

## 1. Introduction

Essential oils are combinations of volatile and odorous compounds that are extracted from different parts of many plant species. The bioactive constituents of EOs have antioxidant, antimicrobial, larvicidal, insect-behavior-modifying, and other properties; consequently, they play an increasing role in the healthcare, cosmetic, food, and agriculture industries [1].

Deadly diseases like malaria, dengue fever, chikungunya, zika, and yellow fever are spread by mosquitoes [2]. Chemical insecticides have been used for decades to control mosquitoes, but their ability to develop resistance to synthetic pesticides has become a major problem, with several cases of pyrethroid, carbamate, organochloride, and organophosphate resistance [3]. Most commercially available insecticides are detrimental to the environment, human health, and non-target organisms. In addition to synthetic insecticides, N, N-diethyl-m-toluamide (DEET)-based mosquito repellents are also used to prevent mosquito bites [4]. However, several studies have reported the harmful effects associated with using DEET-based repellents, including skin irritation, encephalopathy in children, and allergic reactions [5,6]. This predicament has prompted agrochemical businesses and researchers to look for natural compounds and extracts that could serve as viable replacements [7,8]. Plants are considered a potential resource for mosquito control [9] and offer an eco-friendly approach to preventing the spread of mosquito-borne diseases [10,11].

The increasing resistance of pathogens against antimicrobial agents is a major concern in treating infectious diseases [12]. For the treatment of many diseases, plants are the best resource, since they contain numerous metabolites and bioactive constituents that have shown notable effects against pathogenic bacteria (Verma and Singh, 2008). In comparison to synthetic antibacterial compounds, herbal medicines pose fewer adverse effects and have antibacterial potential. Research shows that medicinal plant products are increasingly used to treat bacterial infections [13]. The complex chemical makeup of essential oils makes it more difficult for microbes to generate resistance mechanisms, reducing the possibility of developing resistance [14,15].

*Artemisia* is a genus of the Asteraceae family and is commonly known as sagebrush or wormwood. It is among the most widely distributed genera of Asteraceae, which has about 500 species of herbs and shrubs, found in Asia, Europe, and North America [16,17,18]. *Artemisia* species are famous for their wide range of biological activity. They show various types of pharmacological activity, such as analgesic, antimalarial, tonic, antihelmintic, anti-diabetic, and antipyretic, and also are used in treating wounds, ulcers, tuberculosis, and bronchitis in traditional medicine [17]. Certain species of *Artemisia* are well known for their antimicrobial, antifungal, anti-diabetic, antimalarial, cytotoxic, antioxidant, antipyretic, and insect-repellent activity [19].

*Artemisia scoparia*, commonly known as “red stem wormwood”, is an aromatic and perennial herb that is commonly found in Asia and Europe [17]. Because of the wide distribution of *A. scoparia*, it is a valuable medicinal plant that is rich in phytotoxins and volatile and non-volatile phytochemicals, having medicinal uses [17,18]. *A. scoparia* has many health-promoting outcomes, including liver protection and anticancer, antioxidant, anti-inflammatory, anti-nociceptive, antibacterial, and antipyretic activity [17,20]; besides these, it is also used to treat the common cold and respiratory system disorders and to promote digestion [21].

The diverse biological properties of *A. scoparia* are attributed to the presence of various phytochemicals, including flavonoids, chromones, phenolic glycosides, phenolic acids, steroids, alkynes, coumarins, terpenoids, and volatile constituents [17,22]. In the literature, there are studies on the chemical composition of the essential oils extracted from *A. scoparia* growing in different countries. For example, a study from Iran showed that the major components of the essential oil of *A. scoparia* were 1-phenyl-penta-2,4-diyne, limonene, and β-pinene [23]. Another study from Iran showed that β-thujone, α-thujone, and 1,8-cineole were the major components of *A. scoparia* essential oil [24]. A study from Saudi Arabia revealed that 80% of the *A. scoparia* essential oil comprised two compounds, 2-nonanone and 2-undecanone [25]. Zhigzhitzhapova et al. reported the chemical compositions of essential oils extracted from *A. scoparia* harvested from different districts of Buryatia and Mongolia [21]; however, the literature is lacking on the chemical composition of *A. scoparia* growing at different altitudes. In the proposed study, *A. scoparia* samples were collected from different areas of Pakistan located at different altitudes. Essential oils were extracted through steam distillation and analyzed by GC–MS. The extracted essential oils were further tested for their antibacterial, mosquito-repellent, and larvicidal potential.

## 2. Results

### 2.1. Yields of Essential Oils

The percentage yields of the essential oils extracted from the aerial parts of *A. scoparia* collected from five different areas of Pakistan are shown in Table 1. The highest yield of essential oil was extracted from the *A. scoparia* sample collected from Haripur (0.57 ± 0.04%), whereas the lowest yields were obtained from the samples harvested from Abbottabad and Swat (Table 1).

### 2.2. Chemical Composition of Essential Oils

The GC–MS analysis of the *A. scoparia* essential oils revealed the presence of several different compounds. In all the essential oil samples extracted from *A. scoparia*, the most abundant compounds were capillene (9.6%–31.8%), β-myrcene (6.8%–21.5%), γ-terpinene (9.2%–19.5%), *p*-cymene (3.2%–14.6%), and limonene (4.4%–14.6%) (Table 2). Capillene was observed in all essential oil samples; however, its relative abundance was significantly different (*p* < 0.05) in all samples except Asco-1 and Asco-5, which contained similar (*p* > 0.05) amounts of capillene. Methyleugenol was abundant in the essential oils extracted from the Asco-4 and Asco-5 samples, which were significantly different (*p* < 0.05) from each other, but only a minor proportion was found in the other essential oils. In addition, the oils of Asco-2, Asco-4, and Asco-5 contained large amounts of *trans*-β-caryophyllene, where the relative abondance of this compound was similar (*p* > 0.05) in Asco-2 and Asco-4 (Table 2). β-Acoradiene was present in Asco-2, Asco-3, and Asco-4, where the relative abundance of this compound was significantly higher in Asco-2 (Table 2). Eugenol was not detected in Asco-1 and Asco-2; however, as compared to other samples, it was found in a high percentage in Asco-5 (Table 2).

### 2.3. Antibacterial Activity

*E. coli* was the most susceptible bacterium to all tested essential oils, whereas the PAO1 strain of *P. aeruginosa* was the most resistant among all tested bacteria (Table 3). The most active essential oil was Asco-5 (Table 3).

**Figure 1:**
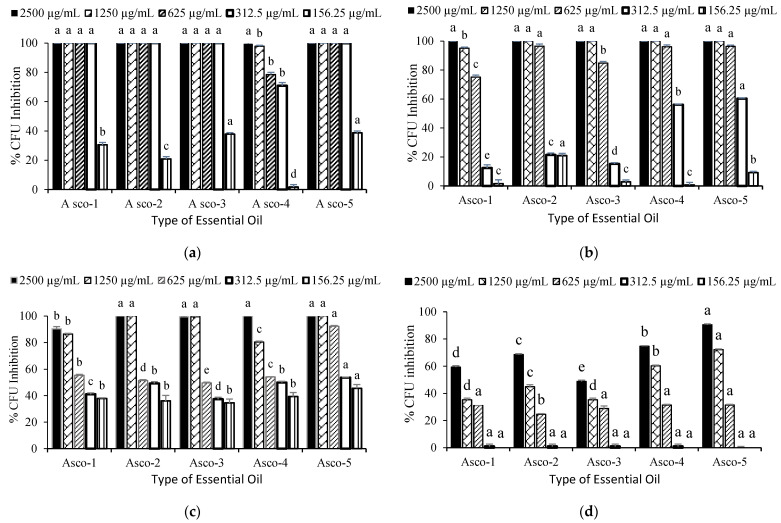
Percentage of bacterial growth inhibition with respect to a negative control of different *A. scoparia* essential oil samples tested at 156–2500 µg/mL against (**a**) *E. coli*; (**b**) *B. subtilis*; (**c**) *S. aureus*; (**d**) *P. aeruginosa PAO1*. Different letters on bars indicate statistical differences (*p* < 0.05, ANOVA post-hoc Bonferroni) in the antibacterial activity of different essential oils tested at the same concentration. Error bars denote standard error *n* = 5.

All the essential oil samples showed significant antibacterial activity against *E. coli*, with 100% bacterial growth inhibition at 625 µg/mL and 312 µg/mL, except for the Asco-4 sample, which showed only 78.5% and 71% CFU inhibition, respectively, which were significantly lower (*p* < 0.05) compared to other essential oil samples at the same concentration (Figure 1a). *B. subtilis* was found to be more resistant compared to *E. coli*; thus, no essential oil exhibited 100% CFU inhibition when tested at 625 µg/mL. At this medium concentration, Asco-2, Asco-4, and Asco-5 showed similar growth inhibition (*p* > 0.05) that was significantly higher (*p* < 0.05) than that of Asco-1 and Asco-3 (Figure 1b). Asco-5 exhibited the highest bioactivity against *S. aureus*, whereas Asco-1 showed the lowest activity among all tested samples. When tested at 625 µg/mL, none of the samples showed 100% growth inhibition; however, Asco-5 exhibited more than 92% inhibition, which was significantly higher (*p* < 0.05) than that of other *A. scoparia* samples (Figure 1c). *P. aeruginosa* (PAO1) was the most resistant among all tested bacterial strains, so none of the *A. scoparia* essential oil samples showed complete inhibition, even at 2500 µg/mL. The most active essential oil sample was Asco-5, which exhibited 90.5% and 72.1% CFU growth inhibition at 2500 µg/mL and 1250 µg/mL, respectively (Figure 1d).

### 2.4. Mosquito-Repellent Activity

**Figure 2:**
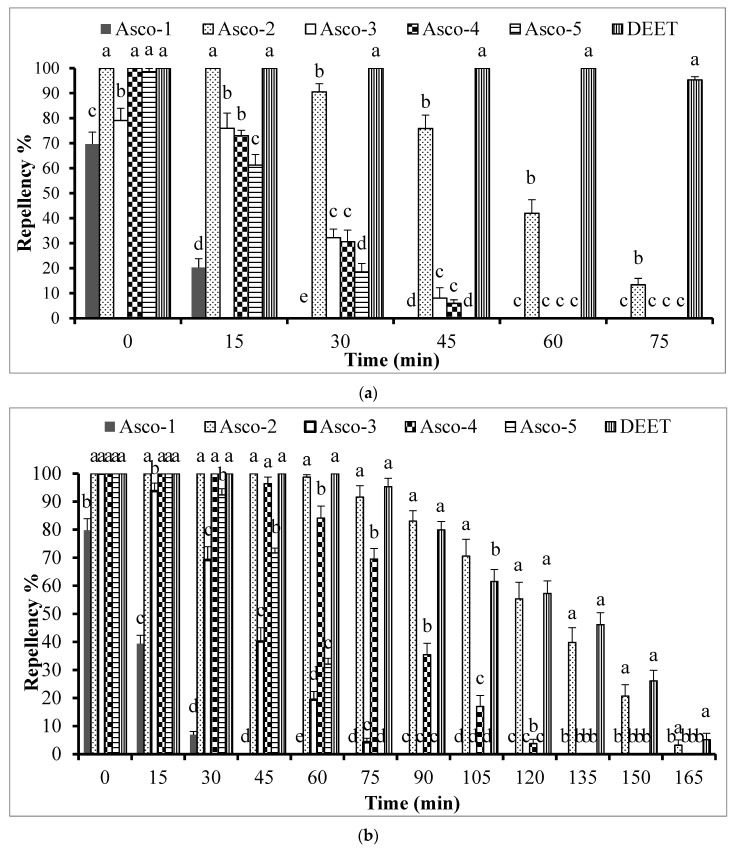
Repellency over time of five *A. scoparia* essential oil samples tested against *Ae. aegypti* females at the doses of (**a**) 33.3 μg/cm^2^ and (**b**) 333 μg/cm^2^. DEET was tested at 33.3 μg/cm^2^ in both experiments. Bars having different letters depict significant differences (*p* < 0.05) in the repellency of test substances examined independently after different time periods (ANOVA post-hoc Bonferroni test). Error bars denote the standard error (*n* = 5).

The *A. scoparia* essential oils showed repellent activity of varying extent against *Ae. aegypti* females at the tested concentrations of 33.3 μg/cm^2^ and 333 μg/cm^2^. The data analysis showed a significant impact of the type of test substance on the repellent activity at 33.3 µg/cm^2^ (df = 5, F = 143, *p* ˂ 0.0001) and at 333 µg/cm^2^ (df = 5, F= 161, *p* ˂ 0.0001). The essential oil sample Asco-2 exhibited the highest repellent activity, whereas Asco-1 showed the lowest activity compared to all other tested essential oil samples (Figure 2). Asco-2 showed 100% repellency towards *Ae. aegypti* for 15 min, which was similar (*p* < 0.05) to that of DEET when tested at 33.3 μg/cm^2^. The repellent activity of both DEET and Asco-2 decreased with the increasing timespan; specifically, DEET and Asco-2 exhibited 95.3% and 13.4% repellent activity at 75 min post-treatment, respectively (Figure 2a).

When increasing the concentration to 333 μg/cm^2^, the bioactivity of all *A. scoparia* essential oils increased but to varying degrees. Asco-2, Asco-3, Asco-4, and Asco-5 exhibited 100% repellency at 60 min, 0 min, 45 min, and 15 min, respectively, whereas Asco-1 showed merely 80% repellency immediately after treatment (Figure 2b). The most active essential oil was Asco-2, which exhibited excellent repellent activity for 165 min. Its activity was similar (*p* > 0.05) to that of a 1% DEET solution (33.3 μg/cm^2^) throughout the testing period (Figure 2b).

### 2.5. Larvicidal Activity

The larvicidal potential of *A. scoparia* essential oil samples was evaluated by the LC_50_ values (Table 4). The most active samples were Asco-5 and Asco-2 as they showed LC_50_ values of 55.5 and 79.3 μg/mL, respectively, for the exposure time of 24 h, while Asco-3 showed the least activity against larvae (Table 4). Upon increasing the exposure time to 48 h, the larvicidal activity of the *A. scoparia* essential oil samples also increased. Asco-5 showed an LC_50_ value of 43.5 μg/mL, which was significantly different (*p* < 0.05) from those of all other samples except Asco-2 (Table 4).

## 3. Discussion

The *A. scoparia* samples, collected from different areas of Pakistan, contained different oil content, with percentage yields of 0.15–0.57%. The highest percentage yield was obtained from the *A. scoparia* sample collected from Haripur (Asco-1), while the lowest oil content was found in the sample harvested from Swat Valley (Asco-2). Interestingly, the plant samples collected at higher altitudes produced lower yields compared to those of lower-altitude areas. The variation in the essential oil content of various plant species is well known [26,27]. Differences in the oil yields of *A. scoparia* were reported in previous studies. For example, a study from India showed that the yield of *A. scoparia* was 0.17% [28], and two other reports from Iran showed that the yield of *A. scoparia* was 0.40% [24] and 0.70% [29]. The differences in oil yield could be attributed to various factors, including the climate of the region, the age of the plant, the nature of the soil, and the collection time, as well as the method of essential oil extraction.

The chemical compositions of essential oils differ from each other with respect to the relative abundance of their chemical constituents. In the present study, capillene was among the most abundant constituents present in all *A. scoparia* essential oils and had the largest relative amounts in the Asco-4 and Asco-5 samples. The γ-terpinene levels were similar in Asco-3 and Asco-5, whereas it was found in lower amounts in the other samples. Overall, capillene (synonym: 2,4-hexadiynylbenzene), methyleugenol, β-myrcene, γ-terpinene, caryophyllene oxide, trans-β-caryophyllene, α-pinene, *p*-cymene, limonene, cis-β-ocimene, and limonene were found as major compounds with different relative abundances. Several previous studies have described the chemical composition of the *A. scoparia* essential oil grown in different countries, but the comparison of the essential oil composition of *A. scoparia* growing at different altitudes and climatic regions is rarely reported. A previous study revealed the chemical composition of *A. scoparia* growing in various districts of Buryatia and Mongolia and described the presence of germacrene D (11.5–40.3%), caryophyllene (4.6–13.8%), caryophyllene oxide (4.3–15.6%), spathulenol (4.0–10.9%), and p-cymene (0.6–15.9%) as major compounds in most of the essential oil samples [21]. A study from Serbia noted the presence of *cis*-β-ocimene (4.3%), γ-terpinene (4.0%), 2,4-pentadienylbenzene (10.0%), eugenol (2.0%), and capillene (63.8%) in the essential oil of *A. scoparia* [30]. Meanwhile, an Iranian study showed that *A. scoparia* growing wild in the Kashan area contained 30.9% 1-phenyl-penta-2,4-diyne (synonym: 2,4-pentadienylbenzene), 23.3% β-pinene, and 10.2% limonene [23]. A study from Tajikistan also reported that the *A. scoparia* essential oil consisted of 2,4-pentadienylbenzene (34.2%), β-pinene (21.3%), methyl eugenol (5.5%), and capillene (4.9%) [31]. *A. scoparia* growing in Turkey contained 15.5% spathulenol, 11.4% caryophyllene oxide, 11.8% 1,2-dehydro acenaphthylene, and 4.6% methyleugenol [19]. A recent study from Crimea reported the presence of 44% capillene, 10.9% scoparone, and 5.7% β-pinene in the ethanolic extract of *A. scoparia* [32]. A comparison of the current study’s results with other published data shows that different chemotypes of *A. scoparia* are present, which are dominated by capillene or agropyrene. Moreover, the chemical composition of *A. scoparia* may vary under different ecological conditions, as they play an important role in the presence of major ingredients in plants’ essential oils.

All of the *A. scoparia* essential oils sampled in this study showed good antibacterial activity against the tested bacterial strains but in a dose-dependent manner. All essential oils showed the highest antibacterial activity against *E. coli*, whereas PAO1 was found to be the most resistant bacterium. *P. aeruginosa* PAO1 might be resistant due to the formation of a microfilm around the cell wall that obstructs the penetration of the sample and its action [33]. *B. subtilis* and *S. aureus* (Gram-positive bacteria) showed almost similar susceptibility towards the essential oils, which was between that of *E. coli* and PAO1. Among all tested essential oils, Asco-5 was the most active, showing the lowest MIC and MBC against the tested bacterial strains. Although capillene and γ-terpinene were the most abundant compounds in this essential oil, its higher antibacterial activity might have been due to the combined effects of other major compounds too. Capillene and γ-terpinene were also present in similar proportions in Asco-3, which possessed lower antibacterial activity compared to Asco-5. In the current study, the MIC values of the *A. scoparia* essential oil samples were much lower than those of 1600 and 3200 µg/mL determined in a previous study from South Korea for the *A. scoparia* essential oil against *S. aureus* and *E. coli*, respectively [22]. Similarly, a study from Turkey showed that the *A. scoparia* essential oil had no activity against *P. aeruginosa*, but a moderate zone of inhibition was determined against *S. aureus* and *E. coli*, when about 20 µL (~20 mg) of pure essential oil was applied to a paper disk [19]. Another report from Saudi Arabia showed that the MIC value of *A. scoparia* was greater than 25 µg/mL for *P. aeruginosa* [25]. In 2023, Nikitin et al. reported an MIC of 4000 µg/mL for an *A. scoparia* ethanolic extract against *B. subtilis*, which is higher than that reported in the current study. A possible reason for the difference in antibacterial activity reported in the previous and current research could be the difference in the chemical compositions of the essential oils. In all the studies cited above, the major compounds of the *A. scoparia* essential oils were different from each other, as well as from the one considered in the current study. Moreover, the susceptibility of the bacterial strains used in various studies could be a reason behind these differences. The *P. aeruginosa* strain PAO1 that we used in our study was very resistant compared to other bacterial stains because even ciprofloxacin exhibited a high MIC and MBC against this bacterial strain.

All the essential oil samples of *A. scoparia* showed mosquito-repellent activity against female adult mosquitoes, but of varying degrees. When tested at the lower dose of 33.3 µg/cm^2^, most essential oils exhibited repellency for a few minutes, whereas only Asco-2 showed repellent activity similar to that of DEET for 15 min. When the applied dose was increased tenfold, the repellency over time also increased for all essential oil samples. The essential oils Asco-2 and Asco-4 exhibited the best mosquito-repellent activity over time, with Asco-4 displaying activity for 120 min and Asco-2 showing repellency for 165 min. Moreover, the observed repellency of Asco-2 was similar to that of the positive control, DEET. The long-lasting repellency of the Asco-2 and Asco-4 essential oil samples could be explained based on their chemical compositions, which were substantially different from those of other essential oil samples. The higher repellency of these two essential oil samples could be attributed to the combination of various compounds, including β-acoradiene and *trans*-β-caryophyllene, whose relative abundances were significantly higher in these essential oils compared to other oils. A recent study showed the moderate repellency of *trans*-β-caryophyllene against *Ae. aegypti* adult females [34]. However, the high repellent activity of *A. scoparia* essential oils could be attributed to the presence of major and minor compounds.

Our results showed that the mosquito repellency of different samples of the same species exhibited differences. The most active essential oil, in terms of mosquito repellency, was Asco-2, which was extracted from a plant population picked from Swat. Interestingly, the essential oils distilled from plant samples collected from high-altitude areas were found to be more repellent than their counterparts collected from lower-altitude areas. The mosquito-repellent activity of the *A. scoparia* essential oil has not been extensively studied, and there is only one previous report that describes the mosquito-repellent activity of this plant’s essential oil against *Ae. aegypti* using a Y-tube olfactometer [35]. The mosquito repellency of different essential oils could be attributed to the presence of a number of major components, such as β-myrcene, *p*-cymene, limonene, γ-terpinene, *trans*-β-caryophyllene, β-acoradiene, and capillene. The different proportions of these compounds could result in varying degrees of repellency towards mosquito females. A previous study reported the repellent effect of *A. scoparia* against stored product pest insects *Callosobruchus maculatus*, *Sitophylus oryzea*, and *Triboleum castaneum* using a fumigation bioassay, where the major compounds were 19.0% β-pinene, 17.5% capillene, 15.1% limonene, and 10.9% β-myrcene [36]. Another study from the Philippines reported effective repellency at 10 and 20 μL (~10 and 20 mg) doses of essential oil from *A. scoparia* leaves against *Ae. aegypti*, tested by using a Y-tube olfactometer [35]. The repellent activity of the *A. scoparia* essential oils reported in the current study (333 µg/cm^2^) was higher than that reported in a previous study where a high dose (10,000 µg) of pure essential oil was tested for repellency.

In the larvicidal bioassay, all *A. scoparia* essential oil samples showed activity against *Ae. aegypti* larvae, but to a different extent. The Asco-5 sample was the most active and its larvicidal activity was statistically similar to that of Asco-2 and Asco-4. In the literature, there are very few studies reporting the insecticidal activity of *A. scoparia* against mosquitoes. The study carried out by Gul et al. (2021) presented the larvicidal activity of chromatographic fractions of an *A. scoparia* leaf hexane extract against the third instar of *Culex quinquefasciatus* and reported that slightly polar fractions were the most active, with LC_50_ values of 22.4–28.7 ppm (µg/mL), whereas non-polar to moderately polar fractions showed activity in the range of 63–271 ppm [37], which is comparable to the data reported in the current study. Intriguingly, Asco-5 was found to be the most insecticidal as well as antibacterial compared to all other *A. scoparia* samples. The combined presence of compounds such as capillene, eugenol, methyleugenol, and *trans*-β-caryophyllene might be responsible for the higher larvicidal and antibacterial potential of Asco-5.

## 4. Materials and Methods

### 4.1. Collection and Maintenance of Plant Material

Fresh aerial parts of wild-grown *A. scoparia* were collected from five different areas in Pakistan (Table 1) with the help of a plant taxonomist, Dr. Abdul Nazir, from the Department of Environmental Sciences, COMSATS University Islamabad, Abbottabad Campus, Abbottabad, Pakistan. The voucher specimen No. CUHA-10 was deposited in the herbarium of the Department of Environmental Sciences, COMSATS University Islamabad, Abbottabad Campus, Abbottabad. Approximately 7 kg of fresh plant material was collected from each collection site. The fresh plant material was homogenized before being subjected to essential oil extraction on the same day of harvesting or stored in a freezer at −20 °C to be used for extraction within 48 h of harvesting.

### 4.2. Extraction of Essential Oils

Steam distillation was used for the extraction of essential oils from the fresh aerial parts of the collected plant material, using a method previously described by Azeem et al. [38]. Briefly, the plant material was cut into small pieces using a sharp cutting blade. Weighed plant material of about 2 kg was subjected to steam distillation in a stainless-steel distillation apparatus. Almost 2 L of distilled water was added to the bottom of the stainless-steel vessel to avoid direct contact with the plant material, which was packed in a meshed container adjusted in the vessel above the water level. The vessel was then heated on an electric hot plate and the resulting steam passed through the plant material, extracting the volatile compounds. The steam was cooled down using a water condenser connected externally to the top of the vessel. The distillate, consisting of plant volatiles and water, was collected in a separating funnel for 3 h. The upper layer of essential oil was decanted and weighed with a digital balance. The percentage yield of the extracted essential oil was calculated by dividing the mass of the essential oil by the mass of the plant material and multiplying it by one hundred. From each *A. scoparia* sample, essential oils were extracted in a triplicate manner. The extracted essential oils were stored in glass vials at −20 °C until used for chemical analysis and bioassays.

### 4.3. Chemical Analysis of Essential Oils by GC–MS

The chemical analysis of the essential oils was performed by using gas chromatography coupled with mass spectrometry (GC–MS). A Hewlett Packard GC–MS system (Agilent Technologies Inc., Santa Clara, CA, USA) was used to analyze the essential oil samples. The GC was equipped with a DB-5 (Agilent Technologies Inc., Santa Clara, CA, USA) capillary column with a 30 m length and 0.25 mm internal diameter. The stationary phase of the GC column was (5%-phenyl)-methylpolysiloxane with a film thickness of 0.25 µm. The parameters of the GC and MS were set as previously reported by Azeem et al. [39]. Briefly, the GC injector was isothermally operated at 225 °C. The initial temperature of the GC oven was 40 °C for 2 min; then, it was raised to 230 °C at the rate of 4 °C/min and was afterwards held isothermally at 230 °C for 5 min. High-purity helium with a constant flow rate of 1 mL/min was used as a mobile phase. Diluted solutions of the essential oil samples were injected with an injection volume of 1 µL in splitless mode, which was set for 30 s. The parameters for the mass spectrometer were as follows: the ion source temperature of the MS was isothermally set at 180 °C, electron ionization was employed at 70 eV and the filament off time was set to 5 min, and the mass spectra scan range was 30–400 *m*/*z*. GC chromatogram peak areas were used to determine the proportion composition of an essential oil sample. The GC retention times of the separated compounds were converted into retention indices by applying the Van den Dool and Kratz formula [40], using the retention times of a series of n-alkanes (C_9_-C_24_) analyzed at the same GC–MS parameters used for the essential oil samples. The identification of the compounds was performed by using both the retention indices and the mass spectra and comparing them with the National Institute of Standard Technology (NIST) 2008 MS library, webbook.nist.gov (accessed on 21 December 2023), and published data [39]. The final confirmation of the compounds was performed via the injection of available standards with the same parameters used for the analysis of the essential oil samples. For each sample, the average data of three replicates are presented.

### 4.4. Antibacterial Activity

Four bacterial strains—two Gram-positive bacteria, *Bacillus subtilis* ATCC 6633 and *Staphylococcus aureus* ATCC 6538, and two Gram-negative bacteria, *Escherichia coli* ATCC 25922 and *Pseudomonas aeruginosa* PAO1—were used to test the antibacterial potential of the *A. scoparia* essential oil samples. The bacterial strains were obtained from the Department of Biotechnology, COMSATS University Islamabad, Abbottabad Campus, Abbottabad, Pakistan. The antibacterial activity of the *A. scoparia* essential oils was determined in two ways, i.e., the determination of the minimum inhibitory concentration (MIC) and minimum bactericidal concentration (MBC), as well as the determination of bacterial growth inhibition. The broth dilution method was used to check the antibacterial potential of the *A. scoparia* essential oil samples [41]. For this, freshly grown colonies of bacteria were suspended in 4 mL of sterilized distilled water and the optical density of the suspension was adjusted to the equivalent of the 0.5 McFarland standard; it contained 10^8^ colony-forming units per mL (CFU/mL). These bacterial suspensions were further diluted in sterilized water to obtain a desired concentration of 10^4^ CFU/mL. The percentage of bacterial growth inhibition of the different essential oils was investigated by using a previous method [42], with some modifications. Briefly, a 10 µL aliquot of the bacterial suspension was mixed with 980 µL of sterilized NB, to which 10 µL of the test substance solution or DMSO was added. DMSO was used as a negative control. Different concentrations of essential oils were prepared in DMSO and were added to the bacterial suspensions. Thus, the final concentrations of the test substances in the test tubes were 156 µg/mL to 5000 µg/mL, having two-fold dilutions in each step. After overnight incubation at 37 °C, a 100 µL aliquot of a sample mixture from each test tube was spread evenly on an NA Petri plate and incubated at 37 °C for 24 h, and the number of bacterial colonies grown on control or test Petri plates was counted. The percentage CFU growth inhibition was calculated by using the formula [number of CFU on DMSO-treated NA Petri plates − number of CFU on test substance NA Petri plates] ×100/[number of CFU on DMSO-treated NA Petri plates].

To determine the MIC and MBC of the test substances, the experiment was repeated as described above except that a negative control consisting of 10 µL of a bacterial suspension mixed with 990 µL of sterilized water was used as a water reference to count the number of CFU originally added in any sample or control test tube. After overnight incubation, a 100 µL aliquot of the mixture from the water reference and sample test tubes was spread evenly on an NA Petri plate and incubated at 37 °C for 24 h. Then, the viable CFU were counted on each Petri plate. If the number of CFU in the test substance was less than or equal to the number of CFU in the reference water control, the concentration was considered as the minimum inhibitory concentration (MIC). If there was no viable CFU in the test-substance-treated Petri plate, the concentration was considered as the minimum bactericidal concentration (MBC). The concentration ranges mentioned above were also used in determining the MICs and MBCs of the test substances. In this experiment, ciprofloxacin was used as a positive control, where two-fold dilutions at 2.44–1250 µg/mL were used. At least five replicates of each concentration of a test or control sample were employed.

### 4.5. Rearing of Ae. aegypti Mosquitoes

An *Ae. aegypti* colony was maintained under laboratory conditions as described earlier [39]. Briefly, the eggs of *Ae. aegypti* were added to distilled water that was maintained in a climate chamber set at 25 ± 2 °C and 80 ± 10% relative humidity for a photoperiod of 12 h:12 h light:dark. Pupae were transferred to a separate plastic container containing distilled water that was placed in a Plexiglas mosquito cage till the emergence of adults. Cotton soaked in 10% sugar solution was placed in the adult mosquito cage as food. The hatched larvae were fed a fish diet (Osaka fish diet, India). The larvae were observed daily and the emerged pupae were transferred to a separate plastic container containing distilled water that was placed in a Plexiglas mosquito cage till the emergence of adults. Cotton soaked in 10% sugar solution was placed in the adult mosquito cage as food. The mated female (4–5 days old) mosquitos were fed the blood of an immobilized pigeon. Then, containers (200 mL) filled with distilled water were placed into the adult cage for egg laying. The eggs were shifted to fresh distilled water in a tray for hatching. The procedure was repeated until the number of larvae and adult mosquitoes was sufficient for the larvicidal and mosquito repellency bioassays.

### 4.6. Mosquito Repellency Bioassay

The repellent activity of the extracted *A*. *scoparia* essential oils was investigated against adult female *Ae. aegypti* by using the human bait method, as previously described [43]. One and ten percent solutions of an essential oil prepared in ethanol were used to test the repellency against *Ae. aegypti*, whereas 1% DEET (Sigma-Aldrich, St. Louis, MO, USA) solution in ethanol was used as a positive control. Briefly, 20 3–4-day-old and 24 h sugar-starved female mosquitoes were released in a separate experimental cage. Before the experiment, the volunteer’s hands were washed with fragrance-free soap and dried. The volunteer wore gloves on both hands that covered the entire hand and arm, except for a circular area of 30 cm^2^ on the dorsal side of both hands. An aliquot of a 100 μL solution of the negative control (ethanol solvent) or test substance was evenly applied on the exposed area of the hand, and, before the experiment, the solvent was allowed to evaporate for 3 min. The hand was exposed to the mosquitoes in the experimental cage for 5 min and the number of successful mosquito landings was counted on the negative-control- or test-sample-treated hand. To check the repellency over time, the bioassay was carried out in the same way as described above, but using the same treated hand after each 15 min period and counting females’ landings for 5 min until the number of mosquito landings on the control and treated hands became equal. The human subjects (volunteers) were informed about the test procedure and consent was obtained before conducting the repellency bioassays. Moreover, permission for the use of human subjects was obtained from the Ethical and Biosafety Committee of Bahauddin Zakariya University, Multan. For each test or control substance, the experiment was repeated five times and new mosquitoes were used in each replicate. The percentage of repellency was calculated by using the formula

% Repellency = [(M_c_ − M_t_)/M_c_] × 100

where M_c_ is the number of mosquito landings on the negative control and M_t_ is the number of mosquito landings on the test-substance-treated hand.

### 4.7. Larvicidal Bioassay

The larvicidal activity of the *A. scoparia* essential oils against *Ae. aegypti* larvae was investigated by following the protocol described in Abbas et al. [11]. Briefly, five larvae of mosquitoes were placed in each well of an ice tray (30 mL capacity) containing about 20 mL water. Different dilutions of the essential oils were prepared in DMSO and a 50 µL solution of essential oil or DMSO was added to each well. The final concentration of the test sample in a well was 25 µg/mL to 400 µg/mL, having twofold dilutions at each step. DMSO was used as a negative control. The larvae were exposed to the test samples for 24–48 h to assess their susceptibility. Fish diet was used as food for larvae during the exposure period. The larvae that did not move after the exposure period were considered dead. The experiment was repeated five times for each test concentration.

### 4.8. Statistical Analysis

To identify the statistical difference between the CFU percentage inhibition and the repellent effect of the essential oil samples, the data were analyzed by one-way ANOVA with the post-hoc Bonferroni test. Probit analysis [44] was used to determine the LC_50_ values of the essential oils regarding their larvicidal activity. The lethal concentration estimates for the tested essential oils were considered significantly different (*p* < 0.05) from that of the baseline essential oil if the confidence limits for the relative median potency ratios did not overlap with the value of 1 [38,45]. The statistical tests, ANOVA, probit analysis, and relative median potency analysis were performed by using the computer software SPSS 20 (IBM, Armonk, NY, USA).

## 5. Conclusions

The current study revealed that the essential oils of *A. scoparia* collected from different altitudes yielded different percentages of essential oils. Capillene was the most abundant compound in Asco-1, Asco-4, and Asco-5, having a relative abundance of 22.8%, 31.8%, and 24.6%. γ-Terpinene and β-myrcene were the most abundant components of Asco-2 and Asco-3, respectively. The essential oil of *A. scoparia* collected from Swat (Asco-2) was the most active as a mosquito repellent, whereas that collected from Attock (Asco-5) was found to be the most mosquito-larvicidal and antibacterial compared to all tested samples. The current study suggests that the altitude and climate have significant effects on the chemical composition of the *A. scoparia* population. Moreover, the essential oils of *A. scoparia* could potentially be used in integrated pest control strategies against *Ae. aegypti* mosquitoes and could be used to treat bacterial infections.

## Figures and Tables

**Table 1:** Description of *A. scoparia* samples and percentage yields of essential oils.

Sample Code	Plant Collection			Yield (%)
	Area	Coordinates	Elevation (m)	
Asco-1	Haripur	33°55′50.6″ N 72°54′04.5″ E	518	0.57 ± 0.04 ^a^
Asco-2	Swat	35°21′42.9″ N 72°36′07.7″ E	1793	0.15 ± 0.01 ^c^
Asco-3	Pindi Bhattian	31°55′10.7″ N 73°26′49.3″ E	199	0.35 ± 0.03 ^b^
Asco-4	Abbottabad	34°07′35.1″ N 73°20′06.4″ E	1300	0.20 ± 0.02 ^c^
Asco-5	Attock	33°47′23.4″ N 72°25′36.1″ E	385	0.44 ± 0.04 ^b^

Data presented are percentage yield ± standard error of the mean (*n* = 3). Different lowercase letters in the yield column indicate significant differences between essential oils extracted from *A. scoparia* samples.

**Table 2:** Chemical compositions of *A. scoparia* essential oils extracted from plant samples collected from different locations in Pakistan.

Compound	RI *	Asco-1 ^‡^	Asco-2	Asco-3	Asco-4	Asco-5
α-Thujene	924	^₸^ 0.1 ± 0.0 a	0.1 ± 0.0 a	0.1 ± 0.0 a	0.0 ± 0.0 b	0.1 ± 0.0 a
α-Pinene	930	7.3 ± 0.3 a	3.0 ± 0.1 c	4.9 ± 0.2 b	1.6 ± 0.1 d	2.1 ± 0.2 d
Sabinen	972	0.5 ± 0.1 a	0.5 ± 0.0 a	0.5 ± 0.1 a	0.2 ± 0.0 b	0.5 ± 0.1 a
β-Pinene	973	0.6 ± 0.1 d	1.2 ± 0.1 c	2.0 ± 0.1 b	2.8 ± 0.1 a	1.5 ± 0.1 c
β-Myrcene	990	21.5 ± 0.7 a	11.1 ± 0.6 b	20.0 ± 0.5 a	6.8 ± 0.3 c	9.7 ± 0.4 b
α-Terpinene	1014	0.0 ± 0.0 d	0.4 ± 0.1 b	0.4 ± 0.0 b	0.2 ± 0.0 c	0.6 ± 0.1 a
p-Cymene	1024	14.6 ± 0.5 a	6.8 ± 0.3 b	7.7 ± 0.3 b	3.2 ± 0.1 d	5.1 ± 0.2 c
Limonene	1032	14.1 ± 0.4 a	5.2 ± 0.2 cd	10.6 ± 0.4 b	4.4 ± 0.2 d	6.1 ± 0.2 c
Eucalyptol	1031		1.2 ± 0.1 a			1.3 ± 0.1 a
*cis*-β-Ocimene	1040	3.3 ± 0.2 d	2.8 ± 0.1 d	7.4 ± 0.2 a	5.8 ± 0.3 b	4.1 ± 0.2 c
*trans*-β-Ocimene	1050	0.9 ± 0.1 c	2.0 ± 0.1 a	1.3 ± 0.1 b	1.7 ± 0.1 a	0.7 ± 0.1 c
γ-Terpinene	1058	10.4 ± 0.4 c	15.8 ± 0.5 b	19.4 ± 0.4 a	9.2 ± 0.3 c	19.5 ± 0.3 a
Terpinolene	1089	0.3 ± 0.0 a	0.3 ± 0.1 a	0.3 ± 0.0 a	0.1 ± 0.0 b	0.3 ± 0.0 a
Linalool	1100	0.1 ± 0.0 a		0.1 ± 0.0 a		0.1 ± 0.0 a
3,4-Dimethyl-2,4,6-octatriene	1132	0.2 ± 0.0 b	0.2 ± 0.0 b	0.4 ± 0.0 a	0.3 ± 0.1 a	0.1 ± 0.0 b
Terpinen-4-ol	1175		0.1 ± 0.0			
α-Terpineol	1188		0.1 ± 0.0 a			0.1 ± 0.0 a
2,4-Pentadiynylbenzene	1285	1.6 ± 0.1 c	6.1 ± 0.2 a	2.7 ± 0.1 b	0.4 ± 0.1 d	0.6 ± 0.1 d
Citronellolacetate	1353	0.1 ± 0.0 a		0.1 ± 0.0 a		0.1 ± 0.0 a
Eugenol	1358			0.1 ± 0.0 c	1.2 ± 0.1 b	8.2 ± 0.3 a
Geranylacetate	1383	0.0 ± 0.0 c	0.1 ± 0.0 b	0.8 ± 0.1 a	0.0 ± 0.0 c	0.1 ± 0.0 b
Methyleugenol	1404	0.2 ± 0.0 d	1.4 ± 0.1 c	0.2 ± 0.0 d	12.7 ± 0.4 a	5.6 ± 0.2 b
*trans*-β-Caryophyllene	1420	0.8 ± 0.1 c	12.31 ± 0.4 a	1.1 ± 0.1 c	12.4 ± 0.3 a	6.6 ± 0.2 b
Cedrene	1449		0.2 ± 0.0 a	0.0 ± 0.0 c	0.1 ± 0.0 b	
α-Caryophyllene	1456	0.1 ± 0.0 c	0.8 ± 0.1 a	0.0 ± 0.0 d	0.7 ± 0.1 a	0.4 ± 0.1 b
β-Acoradiene	1479		10.2 ± 0.4 a	0.3 ± 0.0 c	2.1 ± 0.2 b	
α-Curcumene	1482	0.1 ± 0.0 d	1.7 ± 0.1 a	0.3 ± 0.0 c	0.5 ± 0.1 b	0.1 ± 0.0 d
Capillene	1496	22.8 ± 0.9 b	9.6 ± 0.3 d	18.1 ± 0.5 c	31.8 ± 0.7 a	24.6 ± 0.5 b
β-Cadinene	1524	0.0 ± 0.0 c	0.4 ± 0.1 a	0.0 ± 0.0 c	0.2 ± 0.1 b	0.1 ± 0.0 b
Nerolidol	1565		0.4 ± 0.1 a	0.0 ± 0.0 c		0.1 ± 0.0 b
Spathulenol	1576	0.1 ± 0.0 c	3.1 ± 0.2 a	0.1 ± 0.0 c	0.4 ± 0.1 b	0.2 ± 0.0 bc
Caryophyllene oxide	1582	0.6 ± 0.0 a	0.5 ± 0.1 ab	0.3 ± 0.1 bc	0.2 ± 0.0 c	0.2 ± 0.1 c
β-Eudesmol	1654		0.1 ± 0.0 b		0.6 ± 0.1 a	0.1 ± 0.0 b

^‡^ Asco-1, Asco-2, Asco-3, Asco-4, and Asco-5 refer to *Artemisia scoparia* harvested from Haripur, Swat, Pindi Bhattian, Abbottabad, and Attock, respectively. * RI is the retention index, determined using a (5%-phenyl)-methylpolysiloxane column DB-5; ^₸^ value is the percentage of a compound in an essential oil ± standard error of the mean (*n* = 3); 0.0 ± 0.0 indicates a trace amount. Different lowercase letters followed by values indicate statistically significant differences in the relative abundance of compounds presented in a row (ANOVA post-hoc Bonferroni, *p* < 0.05).

**Table 3:** Minimum inhibitory concentrations (MICs) and minimum bactericidal concentrations (MBCs) of *A. scoparia* essential oil samples and positive control, ciprofloxacin, against pathogenic bacteria.

Test Substance	MIC (MBC) (µg/mL)			
	Gram Positive		Gram Negative	
	*B. subtilis*	*S. aureus*	*E. coli*	*P. aeruginosa PAO1*
Asco-1	625 (2500)	625 (5000)	156 (312)	1250 (>5000)
Asco-2	625 (625)	625 (1250)	156 (312)	1250 (>5000)
Asco-3	625 (1250)	625 (1250)	156 (312)	1250 (5000)
Asco-4	625 (625)	625 (2500)	312 (1250)	1250 (5000)
Asco-5	312 (625)	312 (1250)	156 (312)	1250 (5000)
Ciprofloxacin	9.7 (312.5)	9.7 (312.5)	4.9 (312.5)	19.5 (1250)

**Table 4:** Larvicidal activity of *A. scoparia* essential oil samples against *Ae. aegypti* at the exposure time of 24 h and 48 h.

Essential Oil	Exposure Time (h)	* LC_50_ (95% Fiducial Limit) (µg/mL)	Slope ± SE	Chi-Square (df)
Asco-1	24	127.2 (59.09–253.32) c	2.01 ± 0.58	0.75 (5)
Asco-2		79.3 (45.16–142.79) bc	3.62 ± 1.21	1.38 (5)
Asco-3		275.8 (137.96–602.72) d	1.96 ± 0.56	1.06 (5)
Asco-4		89.1 (50.81–156.60) bc	3.58 ± 1.15	0.24 (5)
Asco-5		55.5 (34.26–105.75) b	6.18 ± 2.51	1.2 (5)
Chlorpyrifos		6.9 (3.08–12.69) a	3.04 ± 1.09	0.61 (5)
Asco-1	48	89.4 (41.98–169.88) c	2.329 ± 0.71	1.02 (5)
Asco-2		60.7 (27.50–112.49) bc	2.78 ± 0.95	1.59 (5)
Asco-3		209.1 (112.64–404.78) d	2.40 ± 0.67	1.76 (5)
Asco-4		85.6 (49.85–150.52) c	3.85 ± 1.27	0.41 (5)
Asco-5		43.5 (23.59–80.75) b	5.63 ± 2.35	0.23 (5)
Chlorpyrifos		4.7 (0.64–8.52) a	2.81 ± 1.19	0.72 (5)

* LC_50_: lethal concentration to kill 50% larvae population. Values with different letters indicate significant differences based on relative median potency analysis of essential oils (Table S1a,b). Lethal doses of essential oils were compared with each other independently at separate exposure times.

## Data Availability

The original contributions presented in the study are included in the article; further inquiries can be directed to the corresponding author.

## Supplementary Materials

The following supporting information can be downloaded at: https://www.mdpi.com/article/10.3390/molecules29061359/s1, Table S1a: Relative median potency estimates of *A. scoparia* essential oils tested for mosquito larvicidal activity after 24 h exposure time; Table S1b: Relative median potency estimates of *A. scoparia* essential oils tested for mosquito larvicidal activity after 48 h exposure time.
