# Supplementary material for: Chemical Composition of Essential Oils from Natural Populations of Artemisia scoparia Collected at Different Altitudes: Antibacterial, Mosquito Repellent, and Larvicidal Effects
Source: Molecules. 2024 Mar 19;29(6):1359. doi: 10.3390/molecules29061359 (PMC10975050; doi:10.3390/molecules29061359)
Supplement: Supplementary file 1 [file molecules-29-01359-s001.zip › molecules-2846313-supplementary.pdf]

Supplementary Material to the manuscript

Amna Parveen, Muhammad Ghazanfar Abbas, Ken Keefover-Ring, Muhammad Binyameen, Raimondas Mozūratis, and Muhammad Azeem

Chemical composition of essential oils from natural population of *Artemisia scoparia* collected at different altitudes: antibacterial, mosquito repellent, and larvicidal effects

Table S1a Relative median potency estimates of *A. scoparia* essential oils tested for mosquito larvicidal activity after 24 h exposure time

Baseline essential oil	Essential oils for comparison	Relative median potency ratio ₸	Confidence Limits	
			Lower Bound	Upper Bound
Asco-1	Asco-2	1.625	.816	3.616
	Asco-3*	.476	.201	.941
	Asco-4	1.448	.730	3.126
	Asco-5*	2.465	1.291	5.810
Asco-2	Asco-1	.615	.277	1.225
	Asco-3*	.293	.100	.642
	Asco-4	.891	.420	1.839
	Asco-5	1.517	.809	3.141
Asco-3	Asco-2*	3.414	1.558	10.011
	Asco-1*	2.100	1.063	4.972
	Asco-4*	3.042	1.421	8.485
	Asco-5*	5.177	2.279	17.389
Asco-4	Asco-2	1.122	.544	2.378
	Asco-1	.690	.320	1.370
	Asco-3*	.329	.118	.704
	Asco-5	1.702	.904	3.635
Asco-5	Asco-2	.659	.318	1.236
	Asco-1*	.406	.172	.775
	Asco-3*	.193	.058	.439
	Asco-4	.588	.275	1.106

Table S1b Relative median potency estimates of *A. scoparia* essential oils tested for mosquito larvicidal activity after 48 h exposure time

Baseline essential oil	Essential oils for comparison	Relative median potency ratio ₸	Confidence Limits	
			Lower Bound	Upper Bound
Asco-1	Asco-2	1.479	.766	3.154
	Asco-3*	.439	.178	.867
	Asco-4	1.057	.535	2.111
	Asco-5*	1.975	1.082	4.295
Asco-2	Asco-1	.676	.317	1.305
	Asco-3*	.297	.100	.637
	Asco-4	.714	.332	1.408
	Asco-5	1.335	.737	2.609
Asco-3	Asco-2*	3.372	1.569	9.957
	Asco-1*	2.279	1.154	5.604
	Asco-4*	2.409	1.189	6.145
	Asco-5*	4.501	2.060	14.585
Asco-4	Asco-2	1.400	.710	3.011
	Asco-1	.946	.474	1.868
	Asco-3*	.415	.163	.841
	Asco-5*	1.868	1.007	4.082
Asco-5	Asco-2	.749	.383	1.357
	Asco-1*	.506	.233	.924
	Asco-3*	.222	.069	.485
	Asco-4*	.535	.245	.993

* indicate essential oils possessed significantly higher or lower toxicity in comparison to the baseline essential oil in a table. The LC_{50} estimates for tested essential oil are significantly different ($P < 0.05$) from the baseline essential oil if confidence limits for relative median potency ratios do not overlap with the value 1.

₸ Relative median potency ratio values > 1 indicate that the compared essential oils are more toxic than their respective baseline essential oil. Relative median potency ratio values < 1 indicate compared essential oils are less toxic than their respective baseline essential oil.
